# Let’s talk about sex: exploring factors influencing the discussion of sexual health among chronically Ill patients in general practice

**DOI:** 10.1186/s12875-022-01660-8

**Published:** 2022-03-19

**Authors:** P. C. Barnhoorn, Inge C. Prins, Hannah R. Zuurveen, Brenda L. den Oudsten, Marjolein E. M. den Ouden, Mattijs E. Numans, Henk W. Elzevier, Gaby F. van Ek

**Affiliations:** 1 Department of Public Health and Primary Care, Leiden University Medical Centre, Albinusdreef 2, Leiden, 2300 RC The Netherlands; 2 Department of Psychiatry, Leiden University Medical Centre, Albinusdreef 2, Leiden, 2300 RC The Netherlands; 3 Department Medical and Clinical Psychology, Tilburg University, P.O. Box 90153, 5000 LE Tilburg, The Netherlands; 4 Research Group Technology, Health & Care, Saxion University of Applied Sciences, P.O. Box 70.000, 7500 KB Enschede, the Netherlands; 5 Department of Urology & Department of Medical Decision Making, Leiden University Medical Centre, Albinusdreef 2, Leiden, 2300 RC The Netherlands; 6 Department of Urology, Leiden University Medical Centre, Albinusdreef 2, Leiden, 2300 RC The Netherlands

**Keywords:** Chronic disease, Multimorbidity, General Practitioner, Sexual dysfunction

## Abstract

**Background:**

Chronic diseases are often associated with sexual dysfunction (SD). Little is known about the practice patterns of general practitioners (GPs) regarding sexual care for chronically ill patients. Therefore, the aim of this study was to examine; to what extent GPs discuss SD with chronically ill patients; the barriers that may stop them; and the factors associated with discussing SD.

**Methods:**

A cross-sectional survey using a 58-item questionnaire was sent to 604 Dutch GPs. Descriptive statistics and associations were used for analysis of the data.

**Results:**

Nearly 58% (*n* = 350) of all GPs approached gave a response and 204 questionnaires were analysable (33.8%). Almost 60% of respondents considered discussing SD with patients important (58.3%, *n* = 119). During the first consultation, 67.5% (*n* = 137) of the GPs reported that they never discussed SD. The most important barrier stopping them was lack of time (51.7%, *n* = 104). The majority (90.2%, *n* = 184) stated that the GP was responsible for addressing SD; 70.1% (*n* = 143) indicated that the GP practice somatic care nurse (GPN) was also responsible. Nearly 80% (*n* = 161) of respondents were unaware of agreements within the practice on accountability for discussing SD. This group discussed SD less often during first and follow-up consults (*p* = 0.002 and *p* < 0.001, respectively). Of the respondents, 61.5% (*n* = 116) felt that they had received insufficient education in SD and 74.6% (*n* = 150) stated that the subject is seldom discussed during training. Approximately 62% of the GPs (*n* = 123) wanted to increase their knowledge, preferably through extra training. According to 53.2% of the GPs (*n* = 107) it was important to improve the knowledge of the GPN. The most frequently mentioned tool that could help improve the conversation about SD was the availability of information brochures for patients (*n* = 123, 60.3%).

**Conclusions:**

This study indicates that Dutch GPs do not discuss SD with chronically ill patients routinely, mainly due to lack of time. An efficient tool is needed to enable GPs to address SD in a time-saving manner. Increased availability of informational materials, agreements on accountability within GP practices, and extra training for the GPs and GPNs could improve the discussion of SD.

## Introduction

The increased prevalence of chronic disease is a great burden for the health care systems worldwide [1]. In the Netherlands, the general practitioner (GP) is the gatekeeper of the health care system and is accountable for the vast majority of chronic disease care [2]. The prevalence of multimorbidity is growing and at the same time, the management of chronically ill patients is shifting from secondary to primary care, resulting in an ever-increasing workload for the GP [2, 3].

Many chronic diseases, including cardiovascular disease, diabetes mellitus and chronic obstructive pulmonary disease (COPD) are strongly associated with sexual dysfunction (SD) [4–8]. Sexual function depends on several different systems, as among which are the vascular and endocrine systems. When chronic disease develops, these systems can be altered due to tissue damage or hormonal changes [9, 10]. These alterations can result in the disruption of the genital response and eventually lead to the development of SD [10]. The most prevalent SDs in men with chronic diseases are decreased sexual desire and erectile dysfunction, which are often medication related. Women with chronic disease often experience a decrease in desire, and pain during intercourse [11–15]. Other diseases causing SD include renal failure, numerous neurological diseases and depression [16, 17].

Since SD often has a significant impact on patients’ wellbeing, adequate sexual care for chronically ill patients is essential in order to improve overall health [18]. Considering that the GP is the first port of call in the Netherlands for medical problems, GPs could be of great importance in the detection of and counseling for SD in patients with chronic diseases [19]. Unfortunately, the literature suggests that GPs do not routinely discuss SD with their chronically ill patients, mostly because of a perceived lack of time [8]. Seen in this light, support from another healthcare professional in providing sexual care might be beneficial. This supportive role in sexual care could be fulfilled by the general practice nurse (GPN): GPNs play an important role in the follow-up of patients in structured chronic disease care [20, 21]. The training for assistant practitioners and nurses to become a GPN was introduced in 1999 by the Dutch government in order to support GPs in providing care for patients with chronic diseases [20]. The GPN’s expertise consists of either somatic care or mental healthcare [21–23]. The GPN mental health-care offers basic psychological guidance. The most important task of the GPN somatic care is to perform routine check-ups in chronically ill patients. The most common chronic diseases seen in GP practice are diabetes, cardiovascular disease and asthma/COPD [24], chronic diseases which are associated with a wide array of SD. A recent study showed that GPNs considered discussing SD with their chronic patients as an important part of their job [21]. However, it also found that GPNs did not discuss SD with chronically ill patients routinely, suggesting this may have been due to a lack of experience and guidelines on SD, insufficient knowledge and training, and reasons related to cultural and ethnic diversity [21].

Little is known about the practice patterns of Dutch GPs regarding sexual care for chronically ill patients, and their perspective on the relatively new role of the GPN in this important aspect of patient care. Because of the significant impact of SD on patients’ well-being, early detection is essential. The GP and GPN can both play an important role in the detection and counseling of SD. Therefore, the aim of this cross-sectional study is to explore the perspective of the Dutch GP on (I) the frequency and importance of discussing SD with their chronically ill patients; (II) barriers stopping GPs from discussing SD with chronically ill patients; (III) the current accountability and management for discussing SD with chronically ill patients; (IV) GPs’ levels of knowledge and competence, and the education they have received in SD in general; (V) tools which might improve discussion of SD with chronically ill patients; (VI) the role of the GPN in the management of SD in chronically ill patients; (VII) factors associated with discussing SD.

## Methods

### Study design

To evaluate the practice patterns of Dutch GPs around the discussion of SD with chronically ill patients, a cross-sectional format was chosen using a questionnaire. In total, 599 questionnaires were sent to the work address of the selected GPs: addresses were obtained from ‘www.zorgkaartnederland.nl’. The Netherlands has approximately 12,000 GPs working in approximately 5,000 general practices [21]. The first 25 GPs listed under each letter of the alphabet were included to ensure randomisation and avoid selection bias. However, less than 25 names were listed for two of the letters. Therefore, a total of 600 GPs were selected. Since one address was invalid, in total 599 questionnaires were sent. Non-respondents received a reminder letter at two and/or three months after the initial mailing.

### Instrument design

The questionnaire was developed by the authors based on literature and expert opinion. Parts of the questionnaire have been used previously in other studies on sexuality [25–30]. Five GPs in the district of Leiden pilot-tested the content of the questionnaire for clarity and linguistics. Since no comments were made, the survey was not adjusted after the pilot phase. The first sheet of the questionnaire offered an opt-out possibility; the remaining 58 items consisted of multiple choice and open-ended questions focusing on:

1. GP demographic and practice characteristics
  Demographic characteristics: age, sex, years of experience, and a degree or training in sexology
  Practice characteristics: the type of practice, number of patients, information about the area in which the practice was located and the absence/presence of a GPN.
2. Discussing SD
  Frequency and perceived importance of discussing SD with patients diagnosed with a chronic disease, during the first and follow-up consult;
  Frequency of chronically ill patients raising the issue of SD spontaneously, combined with whether or not their partner was present;
  Frequency of discussing SD with chronically ill patients in different age and gender categories.
3. Barriers
  Possible barriers that stop GPs from discussing SD with chronically ill patients.
4. Accountability and organisation
  GPs’ perspectives on who is accountable for discussing SD with chronically ill patients;
  Organisation and management of discussions around SD with chronically ill patients within the GP practice.
5. Level of knowledge, competence, and education received
  GPs’ level of knowledge, competence, and education received on SD in general;
  Attention for SD in general during in-service training and GPs’ need to expand their knowledge
6. Tools for improvement
  Tools to improve the discussion of SD with chronically ill patients.
7. Role of the GPN
  The GPs’ perspective on the role of the GPN in providing sexual care for chronically ill patients.

### Statistical methods

Data analysis was performed using SPSS version 23 (SPSS Inc., Chicago, IL, USA). Descriptive analyses of baseline characteristics included calculation of means and standard deviations for continuous variables (i.e. age) and frequencies and percentages for categorical variables. In addition, the median number of patients per practice was calculated. Additional descriptive analyses (frequencies and percentages) were performed to explore the aims of the present study regarding: 1) frequency and importance of discussing SD; 2) barriers; 3) accountability and management; 4) level of knowledge, competence and education; 5) tools for improvement; and 6) role of the GPN. To describe the answers to the question: ‘Are agreements made within the practice on who is accountable for discussing SD with chronically ill patients?’ the answers ‘Do not know’ and ‘No’ were combined to the answer ‘Not aware of these agreements’. The GPs were asked to what extent they agreed with the following statements: ‘The GPN somatic/psychological care is responsible for discussing SD with chronically ill patients’; to describe the answers to this question the answers ‘agree’ and ‘totally agree were combined to the answer ‘agree’ and the answers ‘disagree’ and ‘totally disagree’ were combined to the answer ‘disagree’. The Cochrane-Armitage Trend Tests (Linear-by-Linear Association) was used to calculate possible associations between categorical data. The analysis was specifically focused on factors which had an association with frequency of discussing SD with chronically ill patients during consultations (i.e., years of experience, level of knowledge and being aware of agreement on who is accountable for discussing SD with chronically ill patients). Two-sided *P*-values of < 0.05 were considered statistically significant.

### Ethical considerations

Since this study did not involve patients or interventions, no formal ethical approval is needed in the Netherlands. Written informed consent was obtained from all individual participants included in the study.

## Results

### Response

From the 599 GPs who were approached, 345 returned the questionnaire (57.6%); 199 GPs (33.2%) completed the survey and 143 (23.9%) stated they were not willing to participate. Reasons not to participate were lack of time (*n* = 119), lack of interest (*n* = 25), lack of experience (*n* = 14), and retirement (*n* = 3): some GPs gave multiple reasons for not participating. Three completed questionnaires (0.5%) were excluded because they were completed by a GPN instead of a GP. Since the survey was not changed after the pilot, the five completed pilot questionnaires were included in the analysis resulting in 604 distributed surveys. In total, 204 completed questionnaires of the 604 distributed surveys were analysed (33.8%), however, not every participant answered every question completely, leading to different denominators in the response groups for some questions.

### Demographic information

Demographic characteristics and the description of the GP’s medical practice are presented in Table 1. The respondents consisted of 106 males (52%) and 98 females (48%). The mean age was 49 years (SD = 10.0). About half of the GPs (54.9%, *n* = 112) had more than 15 years of practice as a GP. Almost all GPs had no training in sexology (98.0%, *n* = 197). Ninety-seven percent had a general practice nurse (GPN) working within or in service to their practice (*n* = 195).

**Table 1:** Demographic characteristics and the description of the GP’s medical practice

Respondent characteristics	*N* (%)
Sex (*N* = 204)	
Male	106 (52.0)
Female	98 (48.0)
Age	
Mean (SD)	49.1 (10.0)
Time of practice as a GP (*N* = 204)	
0–11 months	1 (0.5)
1–2 years	3 (1.5)
3–5 years	13 (6.4)
6–10 years	33 (16.2)
11–15 years	42 (20.6)
15 years or more	112 (54.9)
Training in sexology (*N* = 201)	
Yes	4 (2.0)
No	197 (98.0)
Type of practice ^a^	
Solo practice	42 (20.6)
Duo practice	74 (36.6)
Group practice	60 (29.4)
Healthcare Centre	34 (16.7)
Other ^b^	7 (3.4)
Location of the practice (*N* = 200)	
City	96 (48.0)
Village	72 (36.0)
Countryside	15 (7.5)
Other ^c^	17 (8.5)
Number of patients	
Median (min–max)	2825 (280–13,000)
Presence of a GPN ^d^ (*N* = 201)	
Yes	195 (97.0)
No	6 (3.0)

^a^n differs due to multiple answers could be given to this question

^b^ includes answers such as: a duo practice with two days per week three GPs, HOED practice: multiple GPs under one roof, Academic Healthcare Centre

^c^ includes answers such as: village + city, village + countryside, city with 20,000 citizens, urbanised countryside

^d^ includes GPN specialised in mental health and GPN specialised in somatic care

### Discussing SD

The majority of GP respondents considered discussing SD with chronically ill patients to be important (58.3%, *n* = 119) or slightly important (37.7%, *n* = 77). Only 3.4% (*n* = 7) stated that it was *very* important to discuss SD with this patient group. Table 2 shows information about how often GPs discussed SD with chronically ill patients. More than two thirds of GPs *never* discussed SD with their chronically ill patients during a first consult (67.5%, *n* = 137): during follow-up, 53.7% (*n* = 109) of the GPs discussed SD with chronically ill patients. No significant association was found between the experience the GP had and the frequency of discussing SD with chronically ill patients during first (*p* = 0.464) and follow-up consults (*p* = 0.786). Almost half (47.8%, *n* = 97)) of the GPs stated that patients with chronic disease raised SD spontaneously during consultationsin less than half of the cases, while 31.5% said that these patients never raised SD spontaneously (*n* = 64). The majority of respondents reported that they discussed SD in less than half of cases with both male (49.0%, *n* = 99) and female (48.0%, *n* = 97) patients with chronic disease. Of the respondents, 49.7% (*n* = 98) stated that they never discussed sexual dysfunction with chronically ill patients aged 16–35 years and 58.9% (*n* = 116) never discussed this topic with chronically ill patients aged 76 years or older.

**Table 2:** Frequency of SD discussions

How often do you discuss SD with chronic patients during:	Never, *N* (%)	In less than half of cases, *N* (%)	In half of the cases, *N* (%)	In more than half of cases, *N* (%)	Always, *N* (%)
First consult (*N* = 203)	137 (67.5)	58 (28.6)	5 (2.5)	3 (1.5)	0 (0.0)
Follow-up (*N* = 203)	39 (19.2)	109 (53.7)	26 (12.8)	21 (10.3)	8 (3.9)
How often do you discuss SD with chronic patients when:	Never, *N* (%)	In less than half of cases, *N* (%)	In half of the cases, *N* (%)	In more than half of cases, *N* (%)	Always, *N* (%)
Patients don’t raise SD spontaneously (*N* = 203)	64 (31.5)	97 (47.8)	21 (10.3)	19 (9.4)	2 (1.0)
The partner of the patient is present (*N* = 202)	109 (54.0)	80 (39.6)	12 (5.9)	1 (0.5)	0 (0.0)
How often do you discuss SD with chronic disease patients in the following age-groups?:	Never, *N* (%)	In less than half of cases *N*, (%)	In half of the cases, *N* (%)	In more than half of cases *N*, (%)	Always, *N* (%)
16–35 years (*N* = 197)	98 (49.7)	56 (28.4)	17 (8.6)	20 (10.2)	6 (3.0)
36–50 years (*N* = 197)	65 (33.0)	74 (37.6)	34 (17.3)	19 (9.6)	5 (2.5)
51–65 years (*N* = 198)	43 (21.7)	98 (49.5)	34 (17.2)	18 (9.1)	5 (2.5)
66–75 years (*N* = 198)	65 (32.8)	96 (48.5)	21 (10.6)	13 (6.6)	3 (1.5)
76 years or older (*N* = 197)	116 (58.9)	67 (34.0)	10 (5.1)	3 (1.5)	1 (0.5)
Male patients (*N* = 202)	45 (22.3)	99 (49.0)	31 (15.3)	20 (9.9)	7 (3.5)
Female patients (*N* = 202)	59 (29.2)	97 (48.0)	31 (15.3)	9 (4.5)	6 (3.0)

### Barriers

Table 3 details potential barriers to the discussion of SD with chronically ill patients. The barrier most agreed upon was a lack of time (51.7%, *n* = 104). Other important barriers were not being able to find a suitable moment to discuss SD (50.2%, *n* = 101), barriers related to language or ethnicity (43.1%, *n* = 87), and the fact that patients did not spontaneously raise SD during the consult (39.6%, *n* = 80). From the options presented, the barrier mentioned least often was someone else being accountable for discussing SD (1.5%, *n* = 3).

**Table 3:** Barriers to the discussion of SD

Reasons for not addressing SD	Agree ^a^ *N* (%)	Not sure *N* (%)	Disagree ^b^ *N* (%)
Lack of time (*N* = 201)	104 (51.7)	46 (22.9)	51 (25.4)
Could not find a suitable moment (*N* = 201)	101 (50.2)	50 (24.9)	50 (24.9)
Barriers related to language or ethnicity (*N* = 202)	87 (43.1)	45 (22.3)	70 (34.7)
Patients do not raise SD spontaneously (*N* = 202)	80 (39.6)	51 (25.2)	71 (35.1)
Barriers related to culture and religion (*N* = 200)	71 (35.5)	55 (27.5)	74 (37.0)
Presence of a third person (*N* = 200)	69 (34.5)	57 (28.5)	74 (37.0)
Insufficient training (*N* = 202)	67 (33.2)	59 (29.2)	76 (37.6)
Age of the patient (*N* = 202)	57 (28.2)	60 (29.7)	85 (42.1)
Insufficient knowledge (*N* = 201)	51 (25.4)	63 (31.3)	87 (43.3)
I feel uncomfortable to talk about SD (*N* = 199)	31 (15.6)	57 (28.6)	111 (55.8)
Sexuality is not a problem for the patient (*N* = 203)	30 (14.8)	75 (36.9)	98 (48.3)
Sex is private (*N* = 202)	25 (12.4)	43 (21.3)	134 (66.3)
Patient is not ready to discuss SD (*N* = 202)	24 (11.9)	79 (39.1)	99 (49.0)
No connection with the patient (*N* = 201)	24 (11.9)	60 (29.9)	117 (58.2)
Sense of shame (*N* = 202)	23 (11.4)	49 (24.3)	130 (64.4)
Age difference (*N* = 202)	17 (8.4)	34 (16.8)	151 (74.8)
Afraid to offend the patient (*N* = 201)	16 (8.0)	45 (22.4)	140 (69.7)
Patient is of the opposite sex (*N* = 201)	15 (7.5)	30 (14.9)	156 (77.6)
Responsibility of someone else (*N* = 201)	3 (1.5)	29 (14.4)	169 (84.1)

^a^ Agree contains the answers ‘totally agree’ and ‘agree’

^b^ Disagree contains the answers ‘totally disagree’ and ‘disagree’

### Accountability and organisation

Most GPs (90.2%, *n* = 184) felt that they were responsible for addressing SD with their chronically ill patients, while more than half considered that the GPN somatic care (70.1%, *n* = 143) and patient (50.5%*, n* = 103) were also responsible. Other less frequently mentioned options were the GPN psychological care (36.8%, *n* = 75), the patients’ partner (17.6%, *n* = 36) or the GP’s assistant (4.4%, *n* = 9).

Agreements made within the practice between GP and care support workers regarding who is accountable for discussing SD with chronically ill patients were present in 19.9% (*n* = 40) of practices. When agreements were made, they pointed out that the GPN (somatic and/or mental health) (27.5%, *n* = 11), the GP (12.5%, *n* = 5) or the GP and GPN together (20%, *n* = 8) were responsible. Nearly 80% (*n* = 161) of respondents were unaware of such agreements within their practice; they discussed SD with chronically ill patients less often during first and follow-up consult (*p* = 0.002 and *p* < 0.001, respectively). About a quarter of the GPs had information about SD available within the practice to hand out to patients (22.5%, = 46). GPs stated that they referred, on average, 3.6% of their patients with SD to a specialised healthcare professional.

### Knowledge, competence and education received

GPs were asked to rate their own competence and level of knowledge regarding the discussion of SD in general. In total, 82.3% of them felt competent to discuss SD (*n* = 163) and 66.2% (*n* = 131) felt they had sufficient knowledge to address the subject. GPs with more knowledge discussed SD more often with their chronically ill patients during a follow-up consult (*p* < 0.001). No significant difference was found between the level of knowledge and the frequency of discussing SD with chronically ill patients during a first consult (*p* = 0.100). When focussing on education, 61.5% of the GPs (*n* = 116) felt that they had received insufficient education on SD. During GP training, the subject of SD is seldom discussed according to 74.6% of GPs (*n* = 150). Other responses were ‘frequent’ (22.4%, *n* = 45) or ‘never’ (3.0%, *n* = 6).

### Tools for improvement

**Fig. 1:**
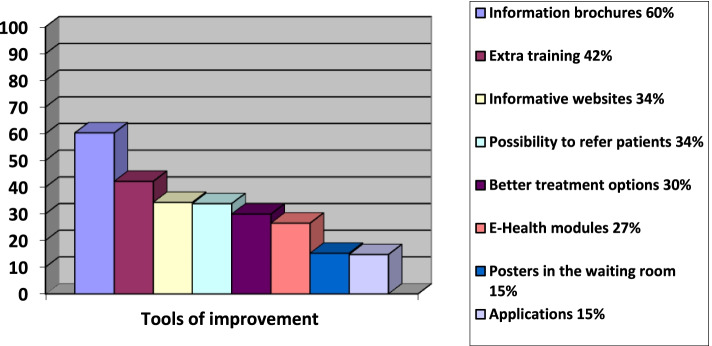
Tools to aid the discussion of SD

Approximately 62% (*n* = 123) of GPs who responded expressed a desire to increase their knowledge about SD in general, preferably through extra training (78.9%, *n* = 97), websites (39.8%, *n* = 49), E-health modules (37.4%, *n* = 46), or applications for mobile phones or tablets (10.6%, *n* = 13). GPs were also asked which tools they felt could help them to improve the conversation about SD with their chronically ill patients (Fig. 1). The most frequently mentioned option was the availability of information brochures for patients (60.3%, *n* = 123). Self-reported answers included for example financial insurance for treatment by a sexologist (3.0%, *n* = 6) and more time available for a consult (1.5%, *n* = 3).

### Role of the GPN 

GPs were asked to what extent they agreed with the following statement: ‘The GPN somatic care is responsible for discussing SD with chronically ill patients’. The majority agreed with this statement (*n* = 152, 76.4%); the remainder either disagreed (17.6%, *n* = 35) or were unsure (6.1%, *n* = 12). The same statement was proposed in relation to the GPN mental health,. In this case, 52.2% agreed (*n* = 104), 36.7% disagreed (*n* = 73) and 11.1% were unsure (*n* = 22). According to 53.2% of the GPs (*n* = 107) it was important to improve the knowledge of the GPN to enable them to discuss SD. Forty per cent thought this was rather important (*n* = 81) and 5.0% (*n* = 10) found it very important. Three GPs did not find this important at all (1.5%).

## Discussion

Dutch GPs feel responsible and competent to discuss SD with their chronically ill patients. Although they considered discussing SD important, this study showed that the majority do not routinely discuss SD with their chronically ill patients, mostly due to lack of time. This is a disturbing finding as many chronic diseases are strongly associated with SD [4–8], the prevalence of chronic disease is rising [1], and the GP plays a major role in the care of chronically ill patients [2, 3]. However, these findings are in agreement with the literature; previous studies have revealed that GPs were afraid SD could not be handled appropriately in the limited time available [19, 31].

Nearly half of the respondents never discussed sexual dysfunction with chronically ill patients aged 16–35 years and over half never discussed the topic with chronically ill patients aged 76 years or older. Whether this suggests that GPs assume younger people are not likely to be affected by SD or that that older people with SD do not need help needs to be explored in future research. The literature however, consistently confirms the value of discussing SD across all age groups [14, 32, 33]. Although GPs report that patients do not raise the subject of SD spontaneously, patients prefer the health professional to bring up the topic [15, 32, 33].

The literature shows that a lack of training in sexology is a barrier for discussion of SD [31]. However, this study found no significant difference between the GPs’ level of knowledge and their frequency of discussing SD with chronically ill patients during a first consult. And, although GPs in this survey rated their knowledge on SD as ‘sufficient’, they rated the education they had received as insufficient, since sexology was seldom discussed during training.

When they were asked if they wanted to improve their knowledge on SD, more than sixty per cent of GPs answered positively, the majority of whom mentioned extra training as a method of doing so. Nevertheless, according to the literature, GPs are afraid extra that training would become time-consuming [34]. Since they are already overburdened with responsibilities, GPs do not have sufficient time for extra activities [34–36]. Given the lack of time most GPs report, it is important that a solution for the improvement of care for chronically ill patients with SD offers tools that enable the GP to discuss the subject in a time-saving manner [19].

Experts in the field have made useful recommendations for discussing sexual health [37]. First, GPs must attempt to secure patients’ trust and openness. Second, questions about sexual health should be asked in a professional and straightforward manner, without losing sight of empathy. To start a conversation about SD, GPs could inform chronically ill patients about illnesses and medications known to have a negative impact on sexual health. In so doing, patients feel they are not alone in suffering from a SD [37]. Then the patients’ medical history can be discussed, and the GP could ask whether, and if so, which SD the patient experiences and whether he or she experiences this as a problem.

Our study suggests additional practical solutions to improve the organisation around sexual health care in chronically ill patients, such as; the availability of informational brochures or websites for patients; extra training for GPs; and the possibility to refer patients to other specialised healthcare providers. This study showed that SD is discussed significantly more often with chronically ill patients when agreements are in place within the GP practice regarding who should take responsibility for those discussions. This represents another practical solution. Furthermore, the literature mentions expanding the role of the GPN as a potential method to improve the management of SD in Dutch GP practices [21, 34]. Indeed, the GPN has a growing role in the care of chronically ill patients and for this reason they could be a very important (and currently underused) resource [21, 38]. Literature suggests that GPs are supportive of the idea of expanding the GPN role in this aspect of care [34]. However, it seems that GPNs are often not involved with the discussion of SD among chronically ill patients partly for same reason as GPs [21, 38]. This indicates that solutions for discussing SD with chronically ill patients in a time-saving manner have to be applicable to both the GP and the GPN in order to improve sexual care for chronically ill patients.

### Strength and limitations

This study is the first to evaluate the practice patterns of Dutch GPs in discussing SD with chronically ill patients: the response rate was 57.6%. However, there could have been a response bias. GPs who responded may be more likely to be familiar with addressing SD with chronically ill patients, or to find the subject of SD important. In addition, the self-reported character of the questionnaire could lead to socially desirable answers. In the present study, a non-validated questionnaire was used, as validated questionnaires did not assess the main objectives of the study. For future purposes, validation of the instrument will be conducted.

## Conclusions

Dutch GPs do not routinely discuss sexual dysfunction with chronically ill patients, mainly due to lack of time. There is need for the implementation of an efficient tool to help GPs and GPNs address SD in a time-saving manner. The availability of informational material and extra training for GPs and their GPNs could improve the discussion of sexual health with chronically ill patients. Support from the GPN and agreements within the practice on accountability for discussing sexual dysfunction further might be beneficial.

## Data Availability

Due to the sensitive nature of the data, information gathered during the current study is available from the corresponding author P.C. Barnhoorn, Department of Public Health and Primary Care, Leiden University Medical Center, Hippocratespad 21, PO box 9600, Zone V0-P, Leiden, 2300 RC, The Netherlands; P.C.Barnhoorn@LUMC.nl on reasonable request to bona fide researchers.

## Abbreviations

COPD: Chronic Obstructive Pulmonary Disease
GP: General Practitioner
GPN: GP nurse
SD: Sexual Dysfunction
